# A BAC-based physical map of the Hessian fly genome anchored to polytene chromosomes

**DOI:** 10.1186/1471-2164-10-293

**Published:** 2009-07-02

**Authors:** Rajat Aggarwal, Thiago R Benatti, Navdeep Gill, Chaoyang Zhao, Ming-Shun Chen, John P Fellers, Brandon J Schemerhorn, Jeff J Stuart

**Affiliations:** 1 Department of Entomology, Purdue University, West Lafayette, Indiana 47907, USA; 2 Department of Agronomy, Purdue University, West Lafayette, Indiana 47907, USA; 3 USDA-ARS, Plant Science and Entomology Research Unit, 4008 Throckmorton, Kansas State University, Manhattan, Kansas 66502, USA; 4 USDA-ARS, Department of Entomology, Purdue University, West Lafayette, Indiana 47907, USA

## Abstract

**Background:**

The Hessian fly (*Mayetiola destructor*) is an important insect pest of wheat. It has tractable genetics, polytene chromosomes, and a small genome (158 Mb). Investigation of the Hessian fly presents excellent opportunities to study plant-insect interactions and the molecular mechanisms underlying genome imprinting and chromosome elimination. A physical map is needed to improve the ability to perform both positional cloning and comparative genomic analyses with the fully sequenced genomes of other dipteran species.

**Results:**

An FPC-based genome wide physical map of the Hessian fly was constructed and anchored to the insect's polytene chromosomes. Bacterial artificial chromosome (BAC) clones corresponding to 12-fold coverage of the Hessian fly genome were fingerprinted, using high information content fingerprinting (HIFC) methodology, and end-sequenced. Fluorescence *in situ *hybridization (FISH) co-localized two BAC clones from each of the 196 longest contigs on the polytene chromosomes. An additional 70 contigs were positioned using a single FISH probe. The 266 FISH mapped contigs were evenly distributed and covered 60% of the genome (95,668 kb). The ends of the fingerprinted BACs were then sequenced to develop the capacity to create sequenced tagged site (STS) markers on the BACs in the map. Only 3.64% of the BAC-end sequence was composed of transposable elements, helicases, ribosomal repeats, simple sequence repeats, and sequences of low complexity. A relatively large fraction (14.27%) of the BES was comprised of multi-copy gene sequences. Nearly 1% of the end sequence was composed of simple sequence repeats (SSRs).

**Conclusion:**

This physical map provides the foundation for high-resolution genetic mapping, map-based cloning, and assembly of complete genome sequencing data. The results indicate that restriction fragment length heterogeneity in BAC libraries used to construct physical maps lower the length and the depth of the contigs, but is not an absolute barrier to the successful application of the technology. This map will serve as a genomic resource for accelerating gene discovery, genome sequencing, and the assembly of BAC sequences. The Hessian fly BAC-clone assembly, and the names and positions of the BAC clones used in the FISH experiments are publically available at .

## Background

The Hessian fly (*Mayetiola destructor*) is an important pest of wheat (*Triticum *spp.) and a member of one of the largest and most economically important families of insects, the gall midges (Cecidomyiidae). Its genetic tractability and short generation time (~28 days) make it especially attractive as an experimental model for investigating insect-plant interactions [1]. This capacity was first demonstrated when it was the first insect shown to have a gene-for-gene interaction with its host plant [2]. Previously, it was used to study chromosome elimination and the function of the germ-line-limited "E" chromosomes that characterize all gall midge species [3-5]. Later investigations demonstrated that it has other biological attributes that are compatible with genomic analyses, including polytene chromosomes in the larval salivary glands [6] and a small genome (158 Mb) [7]. The development of a physically anchored genetic map [8], a syntenic analysis of a BAC-based contig [9], and transcriptomic analyses of the first instar salivary glands [10,11] also demonstrated the potential of this insect for comparative genomics with other dipteran species.

Improved genomic maps are essential to further develop this insect as an experimental organism. However, certain biological characteristics make fine-scale genetic mapping in the Hessian fly problematic. The first problem is the relatively low number of offspring produced by single females, typically ranging from 50 to 200, which limits the resolution of a genetic map. The second problem is the small size (2 to 3 mm in length) of the insect. This severely limits the yield of the genomic DNA extracted from individuals, and the number of molecular markers that can be genetically mapped. A third problem, the insect's unusual chromosome cycle and mechanism of sex determination, makes the construction of inbred strains difficult. Because this phenomenon is both central to the genetics of the insect and worthy of genomic investigation, it is briefly described below.

The germ line of the Hessian fly contains both maternally and paternally derived copies of each of two autosomes (A1 and A2) and two X chromosomes (X1 and X2) [12]. It also contains a variable number (~32) of maternally inherited germ-line-limited "E" chromosomes (A1A2X1X2/A1A2X1X2; E). The E chromosomes are eliminated from all future somatic cells during the fifth cleavage division of embryogenesis [4]. This post-zygotic division also determines sex. Embryos that eliminate the paternally inherited X chromosomes, together with the E chromosomes, possess a male determining somatic karyotype (A1A2X1X2/A1A2OO) and develop as males. Those that retain both sets of X chromosomes possess a female determining somatic karyotype (A1A2X1X2/A1A2X1X2) and develop as females. Maternal genotype determines whether the paternally derived X chromosomes are eliminated or retained in the soma [13]. As a consequence, an unusual mating structure is established that is resistant to inbreeding; females produce either all-female or all-male families. Chromosome elimination also occurs during spermatogenesis. The result is that all sperm carry only the maternally derived autosomes and X chromosomes (A1A2X1X2). Genetic recombination between the homologous autosomes and X chromosomes occurs only during meiosis of oogenesis. All ova carry a single copy of each autosome and each X chromosome and a full complement (~32) of E chromosomes (A1A2X1X2; E). There is no heterogametic sex since all sperm and all ova are genetically equivalent and sex is determined after fertilization.

In contrast to the problems that complicate genetic analyses, the Hessian fly polytene chromosomes make physical mapping relatively simple. Although they lack the banding resolution of *Drosophila *polytene chromosomes, they still provide a relatively high mapping resolution when bacterial artificial chromosomes (BACs) are used as probes for fluorescence *in situ *hybridization (FISH) [8]. This facility suggested that the development of a dense physical map of the Hessian fly's polytene chromosomes should precede high-resolution genetic mapping. We therefore applied the high-information content fingerprinting (HICF) method developed by Luo et al. [14] to construct a BAC contig map using three Hessian fly BAC libraries (Table 1). The result was a first generation FPC-based physical map of the Hessian fly genome that is anchored to the polytene chromosomes. It is based on restriction fingerprints of 13,614 BAC clones. The largest 266 contigs were positioned on the chromosomes by fluorescence *in situ *hybridization (FISH). These contigs contain 4,563 BAC clones, and cover ~60% of the genome. The BAC end sequences (BES) of the clones used in this analysis were determined to provide the capacity to develop sequenced tagged site (STS) markers evenly distributed over the genome. BAC-end sequence data were also analyzed to improve our understanding of Hessian fly genome organization.

**Table 1:** BAC libraries fingerprinted for the Hessian fly physical map

	**BAC Library**		
	
	**Mde**	**Hf**	**CL (MD_Bb)**
**DNA source**	Population vH13	Population GP	Population L
**Vector**	pIndigoBAC	pECBA1	pIndigoBAC536
**Cloning site**	*Hin*dIII	*Hin*dIII	*Bst*yI
**Mean Insert Size (kb)**	55	150	130
**No. FP BAC clones**	78	5,766	6,108
**Mean no. bands/clone**	NA	90.6	87.6
**Genome coverage**	0.006	5.5	5.0

## Results and discussion

### BAC fingerprinting

We subjected 13,614 BACs to high-information content fingerprinting (Table 2). The DNA fingerprint profile of each BAC clone was then converted into an FPC compatible format using the FP Miner 1.1 software (BioinforSoft LLC, OR). The standard deviation associated with the size of two vector bands present in the fingerprints of each BAC (157.4 ± 0.01 bp and 369.5 ± 0.02 bp) indicated that fragment size reproducibility was high [15]. The FP Miner software was used to remove background signals and vector bands, and to assign a quality score to each BAC DNA fingerprint. We examined all of the fingerprints and removed 1662 that failed to meet each of the following quality standards: 1) 30 to 250 fragments, 2) = 10 fragments of each of the four colors, 3) a size standard match quality score that was >0.8, and 4) a fingerprint editing quality score that was >10. The fingerprints of 11,952 BACs (88% of the total) remained available for FPC assembly. The percentage of successfully fingerprinted clones compared favorably with the HICF maps developed for maize (*Zea mays*; 87%) [15], Nile tilapia (*Oreochromis niloticus*; 87%) [16], channel catfish (*Ictalurus punctatus*; 91.5%) [17], and *Brassica rapa *(94.2%) [18]. DNA fragment sizes ranging from 50 to 500 bp were imported into the FPC v8.5.1 [19,20] to build contigs. Among the successfully fingerprinted clones, 5,766 were derived from the Hf library, 6,108 were derived from the CL library, and 78 were derived from the Mde library. BAC clones from the Hf library had an average insert size of 150 kb and the CL library had an average insert size of 130 kb. The average number of scored bands per clone in the Hf library (90.6) and the CL library (87.6) was slightly less than the average observed during the construction of the maize map (107) [15], but slightly greater than the averages of the two BAC libraries used to construct the Nile tilapia map (54 and 70) [16]. Combined, the BACs in the Hf and CL libraries provided an estimated 10.5-fold coverage of the Hessian fly genome (Table 1). This was greater coverage than that used to construct either the Nile tilapia (5.6-fold) [16] or the catfish (6.8-fold) [17] maps, but considerably less than the coverage used to construct the maps of *B. rapa *(15.2-fold) [18] and maize (22-fold) [15].

**Table 2:** Summary of the Hessian fly physical map

**Number of processed clones**		**13,614**
Hf library	6,144	
CL library	7,392	
Mde library	78	
**Number of clones used in contig assembly**		**11,952**
Hf library	5,766	
CL library	6,108	
Mde library	78	
**Number of singletons**		**4,236**
**Number of contigs**		**1,377**
2–4 clones	913	
5–9 clones	275	
10–25 clones	159	
26–50 clones	28	
51–73 clones	2	
**Physical length of the contigs (kb)**		**283,555**

### Contig assembly

Contigs were assembled from the fingerprint data using the computer program FPC version 8.5.1 [15,19,20]. FPC parameters were adjusted for the HICF technique as described by Lou et al. [14] and Nelson [15]. In each assembly, we used a tolerance (5) that restricted two bands with lengths greater than 0.5 bp from being considered the same band. In the initial assembly, we used a cutoff (the threshold for the probability score that matching bands are a coincidence) of 1*e*-35. Using these settings, FPC built 1258 contigs containing from 2 to 126 BAC clones per contig. The DQer function was then used to identify contigs with ≥ 10% questionable clones (Qs). These contigs were then gradually split using three more stringent cutoffs (1*e*-38, 1*e*-41, and 1*e*-44). This produced 1477 contigs containing from 2 to 67 BAC clones per contig. The assembly was then end merged at a cutoff of 1*e*-29. This produced the first generation HICF Hessian fly map, which consisted of 1377 contigs, containing 7,716 BACs, and 4,236 (35%) singletons. The map had an average of 5.6 BACs per contig and the largest contig contained 73 BACs (Table 2). FPC analysis estimated that the clones used to produce the map had 1,061,478 unique bands, averaging 88.8 bands per clone. We used the known lengths of 23 BAC clones and the total number of labeled fragments for each of these clones to estimate average band size (1182 bp). Using this value, the assembled contigs had an estimated length of 283,555 kb, providing approximately 1.8-fold coverage of total genome length. The average contig length was 206 kb and the longest contig covered 1166 kb of the genome. FPC identified a total of 441 questionable clones (Qs) in the assembly. There were 88 contigs with >10% Qs. However, the average number of Qs per contig was only 0.32. Furthermore, 1212 contigs (88%) had no Qs, no contig had more than 14 Qs, and the majority of the Qs were in a few large contigs.

Compared to the contigs of other HICF maps, the Hessian fly contigs were relatively small. For example, the *B. rapa*, catfish, and Nile tilapia maps each had greater average numbers of BACs per contig (37.7, 23.4, and 9.0 respectively), greater average contig lengths (512 kb, 521 kb, 390 kb) and lower percentages of singletons (20.7, 2.0, and 7.5) [16-18]. Since both BAC coverage and fragment size reproducibility were good, other factors were probably responsible for the shorter and shallower contigs in the Hessian fly map. The organization of the much smaller Hessian fly genome might have been one factor. However, we suspect that a greater frequency of restriction fragment length polymorphisms (RFLPs) in the Hessian fly BAC libraries was the major problem. This had been anticipated because the Hessian fly BAC libraries were each constructed with DNA derived from thousands of individuals in heterogeneous strains that are poorly characterized. Thus, indels, single nucleotide polymorphisms, gene duplications, and other rearrangements may all increase the number of mismatched bands. Regardless of the cause and in spite of the relatively low number of BACs per contig, the coverage of total genome length (1.8) appeared to exceed that of the maps of *B. rapa *(1.3), catfish (0.93), and Nile tilapia (1.65). In addition, the percentage of Qs in the Hessian fly assembly (0.32) was lower than that observed in maize (11.0) [15], *B. rapa *(15.0), catfish (7.3), and Nile tilapia (9.6). Thus, it appeared that the Hessian fly contigs provided reasonable coverage with few questionable clones. This suggested that an abundance of RFLPs is not an absolute barrier to the construction of a HICF-based physical map.

Because the BAC clones were largely derived from two different libraries (Table 1), we performed an FPC assembly of each library separately using the same parameters that were used to assemble both libraries combined. The BACs in the CL library assembled into a greater number of contigs (888 vs. 795) with a greater average number of BACs per contig (4.74 vs. 3.78) than the BACs in the Hf library. In addition, the percentage of singletons in the CL assembly (31%) was fewer than the singletons in the Hf assembly (48%), but the number of contigs with >10% Qs was nearly the same (CL = 33 and Hf = 30). Thus, it appeared that if RFLPs were interfering with contig assembly, they were more abundant in the Hf library. Interestingly, the sum of the total number of contigs assembled using the CL (888) and Hf BACs (793) separately was only 22% greater than the total number of BACs in the combined assembly (1377), and the sum of the coverage provided by the CL (159,451 kb) and Hf BACs (149,099 kb) was only 9% greater than that of the combined assembly (283,555 kb). Thus, it appeared as if the contigs of one library only occasionally overlapped with the contigs of the other library and this lowered contig size more than total coverage when the libraries were combined. This possibility was also evident in the contigs of the FPC map where CL BACs tended to stack with other CL BACs and Hf BACs tended to stack with other Hf BACs [21]. It is also consistent with the suggestion that using libraries prepared with different restriction enzymes reduces the number of gaps in the assembly [22-24].

### Contig validation

**Figure 1:**
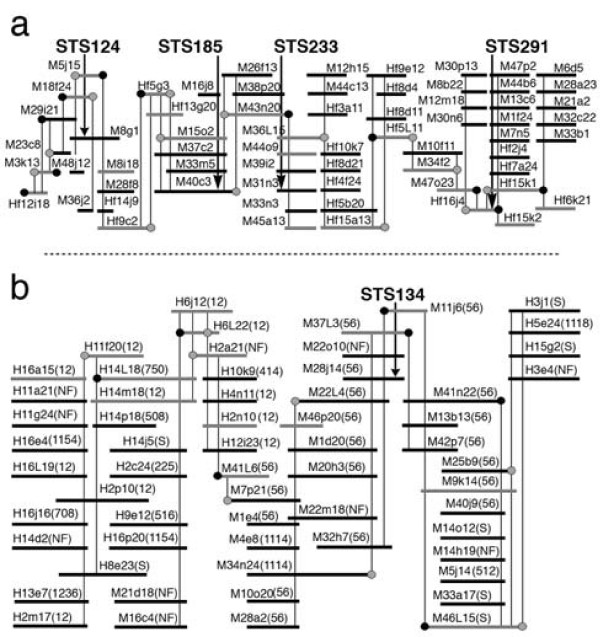
**BAC contigs established by chromosome walking**. BAC clones are represented as black and grey horizontal lines and BES used as probes are represented as black (SP6-end) and grey (T7-end) circles. The physical positions of BAC clones represented by grey lines were determined in FISH experiments. The number and library origin of each BAC clone is shown where M indicates the Mde library, and H indicates the Hf library. The FPC-based contig of each BAC is shown in parenthesis (NF indicates a clone that was not fingerprinted). Black vertical arrows intersect BAC clones that were positive in library screens that used STS markers as probe. Grey vertical lines intersect BAC clones that were positive in library screens that used BES as probe. (a) Chromosome walk-124. (b) Chromosome walk-134.

Two different approaches were used to validate contigs in the first generation Hessian fly HICF map. The first examined the FPC assembly of two contigs that had been constructed by chromosome walking (Figure 1). This was the only region of the Hessian fly genome in which chromosome walking had been previously performed. The construction and composition of one walk (walk-124) was described previously [9]. It consisted of 64 BAC clones covering a 7.5-cM genetic distance that was marked with four STS markers and a physical distance of nearly 1 Mb. The other chromosome walk began from STS marker 134 (walk-134) and was composed of 60 BAC clones. Together, the contigs constructed by walk-124 and walk-134 contained 73 Mde BAC clones that were analyzed by FPC. The 134-walk was deeper and contained a greater proportion of Hf clones. Neither chromosome walk contained clones from the CL library because that library had not yet been prepared when the walking experiments were performed. Only 5 clones in the 124-walk were discovered in the contigs of the FPC assembly (Figure 1). Those clones were present in 3 small contigs (contigs #837, #938, and #1176) containing a combined total of 7 clones. FPC assembly of the 134-walk was more successful. FPC assembled 52 (87%) of the clones in the 134-walk into six contigs (Figure 1). The majority of those clones were assembled into two contigs: one (contig #12) containing 24 134-walk clones and the other (contig #56) containing 19 134-walk clones. An additional 28 CL clones were identified in these contigs. Reducing the stringency of FPC assembly (cutoff of 1*e*-15) merged all six contigs associated with walk-134 into a single contig. However, the assembly of the 124-walk was not improved. This analysis suggested that, as expected, regions of the genome with relatively deep coverage (walk-134) were well represented in the map whereas regions with less depth (walk-124) were poorly represented.

**Figure 2:**
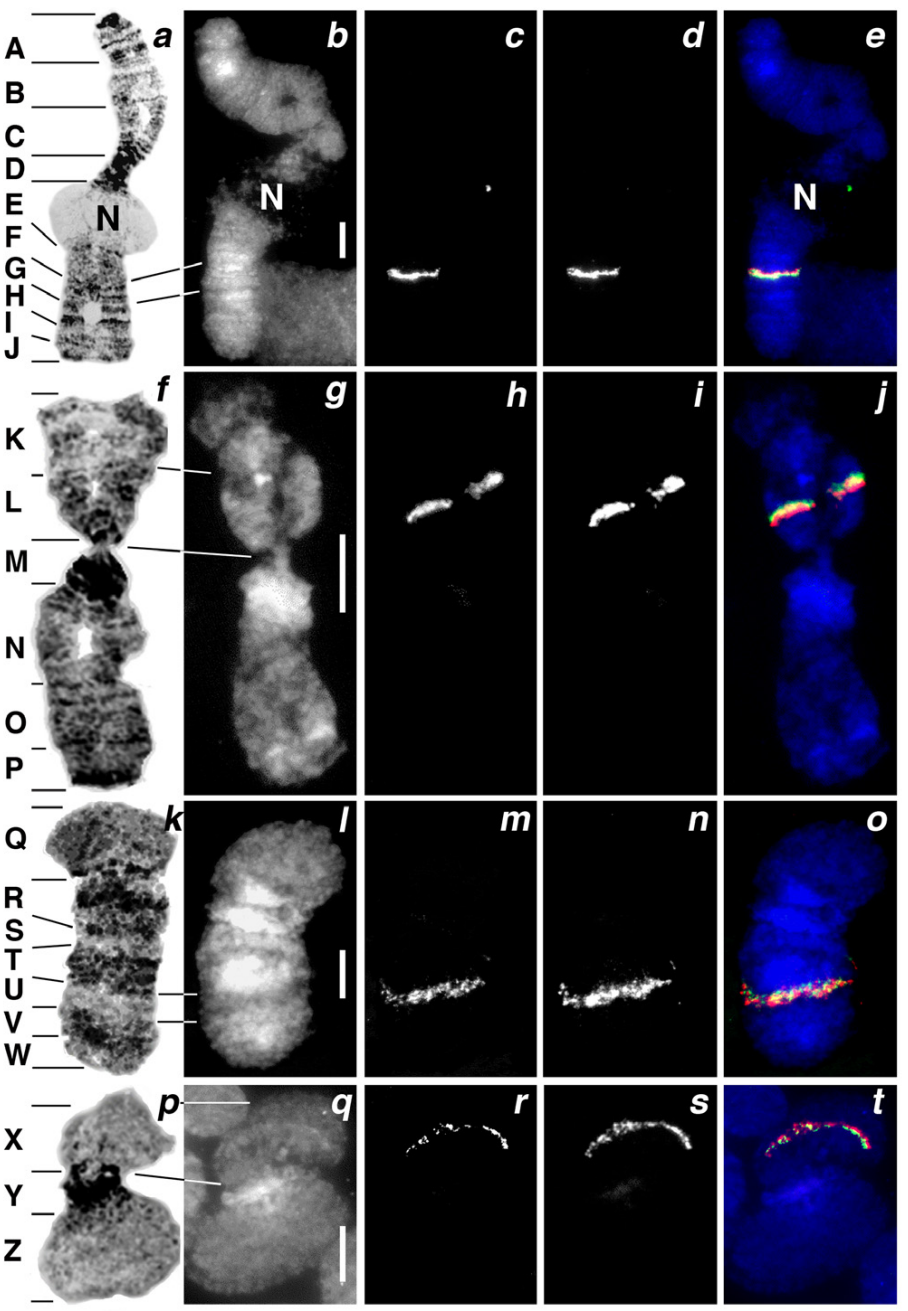
**Hessian fly polytene chromosomes and BAC clone co-localizations**. Chromosome segments (A-Z) are indicated on geimsa stained chromosomes (a, f, k, and p). White bars (10 μm) indicate the relative lengths of corresponding DAPI stained chromosomes (b, g, l, and q). Four examples of co-localizing BAC clones are shown in color overlay images (e, j, o, and t). Contig #320 BAC clones CL4h4 (c) and CL31e21 (d) hybridized to chromosome A1 segment G (e). Contig #199 BAC clones Hf30g15 (h) and Hf4e2 (i) hybridized to chromosome A2 segment L (j). Note that Hf30g15 hybridization (green) was more distal than Hf4e2 hybridization (red), allowing contig orientation. Contig #320 BAC clones CL25e22 (m) and Hf10j14 (n) hybridized to chromosome X1 segment U (o). Contig #320 BAC clones CL29m24 (r) and Hf10j14 (s) hybridized to chromosome X2 segment X (t).

**Figure 3:**
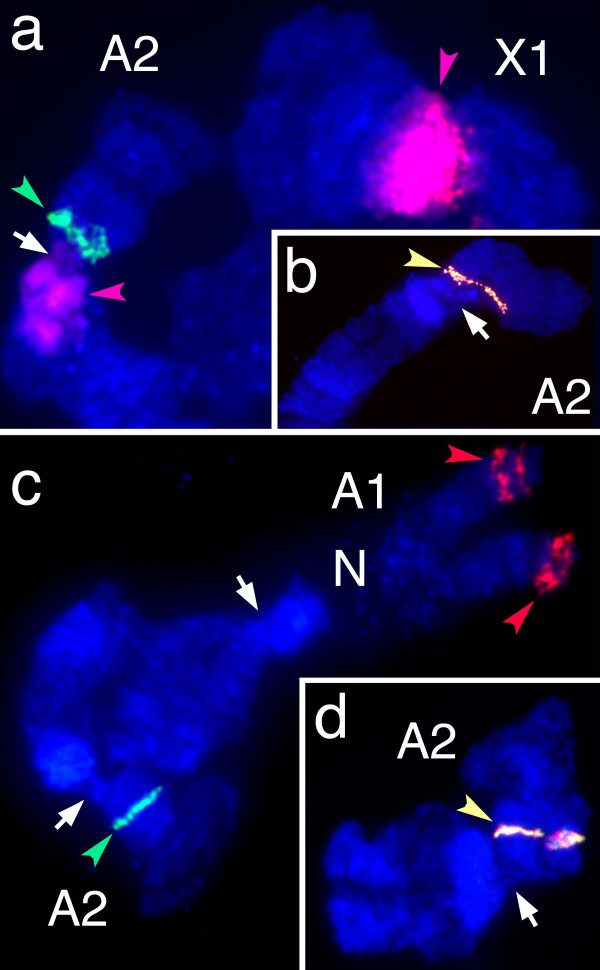
**Resolving "invalid" contigs using FISH**. (a) The hybridization of BACs CL24B4 (green arrow, A2 segment L) and Hf15M12 (red arrows, pericentromeric heterochromatin) defined contig #36 as invalid and repetitive. (b) The hybridization of a third contig #36 BAC (CL24N4, yellow arrow) resolved the position of contig #36 to A2 segment L. (c) The hybridization of BACs CL17J4 (green arrow, A2 segment L) and Hf7B18 (red arrows, A1 segment J) defined contig #234 as invalid but non-repetitive. (d) Hybridization of a third contig #234 BAC clone (Hf13K2, yellow arrow) with BAC clone CL17J4 resolved the position of contig #234 to A2 segment L.

The second approach of assessing contig validity was to test the continuity of the 196 longest FPC assembled contigs using FISH. In these experiments, a BAC clone at one extreme of each FPC assembled contig was labeled with biotin and a BAC clone at the opposite extreme was labeled with digoxigenin. Separate FISH experiments were performed for each contig, in which both BAC clones were hybridized to the same polytene chromosome preparations (Figure 2). The FPC assembly was judged "valid" when both probes from the same contig co-localized on the Hessian fly polytene chromosomes (Table 3). The contigs examined contained from 9 to 73 BAC clones per contig and ranged in length from 172 to 1157 kb. Using the banding patterns of the four polytene Hessian fly chromosomes, we divided the Hessian fly genome into 26 chromosomal segments (Figure 2), and determined which segments contained each valid contig (Table 4). Among the 196 contigs tested, 169 contigs (86%) were scored as valid (Table 3). In 63 of these valid contigs (32% of the total) it was possible to discern which of the two BAC clones was the most proximal (Figure 2). It was, therefore, possible to suggest the orientation of those 63 contigs on the chromosomes. There were 27 contigs (14%) that were determined to be "invalid." These had a greater number of Qs than the valid contigs (Table 3). Moreover, they were easily separated into two groups: A "repetitive" group of 11 contigs, and a "non-repetitive" group of 16 contigs. The contigs in the repetitive group had at least one probe that hybridized to multiple locations (Figure 3). Seven of these had a probe that hybridized to pericentromeric heterochromatin. The non-repetitive group had probes that hybridized to different, but unique, genomic locations. This group of contigs had a greater number of Qs per contig than both the valid group of contigs and the invalid-repetitive group of contigs (Table 3). We reexamined all invalid contigs by performing FISH with additional BAC clones selected from the same contigs. In each experiment, these clones hybridized in the same position as one of the previous BAC clones (Figure 3). These data were then used to assign the unreliable contigs to a chromosomal segment (Table 4).

**Table 3:** Assessment of 196 contigs using two BAC clones per contig as FISH probes.

	**Valid**	**Invalid**	**Invalid-R**	**Invalid-NR**
**Total no. contigs**	169	27	11	16
**Total no. BACs**	3208	575	186	389
**Mean no. BACs/contig**	19 ± 10	21 ± 11	17 ± 11	24 ± 10^*†^
**Total no. Qs**	641	161	41	120
**Total Qs/BAC**	0.20	0.28	0.22	0.31
**Mean no. Qs/contig**	3.8 ± 4.7	6.0 ± 5.3*	3.7 ± 5.7	7.5 ± 4.6^**†^
**Mean no. Qs/BAC/contig**	0.16 ± 0.13	0.25 ± 0.17**	0.15 ± 0.16	0.31 ± 0.15^**††^
**Total CB units** **(estimated kb)**	58,255 (68,857)	10,214 (12,072)	3,563 (4,211)	6,651 (7,861)
**Mean CB units/contig** **(estimated kb/contig)**	344 ± 129 (407 ± 152)	378 ± 148 (447 ± 175)	324 ± 126 (383 ± 150)	415 ± 154* (491 ± 182)

Contigs in which the probes co-localized were assessed as valid. Contigs in which the probes failed to co-localize were assessed as invalid. Invalid contigs were further divided into contigs that either had one or more repetitive BAC probes (Invalid-R) or no repetitive BAC probes (Invalid-NR). *Means significantly different from the Valid mean (Student's *t*; p < 0.10). **Means significantly different from the Valid mean (Student's *t*; p < 0.05). ^†^Means significantly different from the Invalid-R mean (Student's *t*; p < 0.10). ^††^Means significantly different from the Invalid-R mean (Student's *t*; p < 0.05).

**Table 4:** Distribution of 266 FISH-positioned contigs in 26 Hessian fly chromosomal segments (A-Z)

**Chromosome segment**	**% Relative length**	**No. (%) of contigs**	**No. (%) of BAC clones**	**No. (%) of Qs**	**contig length (% of total)**
A*	5.3	23 (8.6)	374 (8.2)	64 (7.3)	8,642 (8.8)
B	4.1	15 (5.6)	249 (5.5)	53 (6.0)	5,929 (6.0)
C	8.1	19 (7.1)	282 (6.2)	49 (5.6)	6,058 (6.2)
D	3.3	5 (1.9)	51 (1.2)	8 (0.9)	1,176 (1.2)
E	0.9	0	0	0	0
F*	5.3	23 (8.6)	460 (10.1)	118 (13.4)	9,145 (9.3)
G*	2.6	7 (2.6)	88 (1.9)	17 (1.9)	2,412 (2.5)
H	1.9	10 (3.8)	183 (4.0)	31 (3.5)	4,285 (4.4)
I	1.7	4 (1.5)	57 (1.2)	9 (1.0)	1,209 (1.2)
J*	2.9	6 (2.3)	185 (4.0)	45 (5.1)	3,059 (3.1)
K*	4.3	13 (4.9)	281 (6.2)	76 (8.6)	6,142 (6.3)
L*	5.3	13 (4.9)	196 (4.3)	41 (4.6)	4,871 (5.0)
M*	2.6	1 (0.4)	10 (0.2)	0	314 (0.3)
N*	6.7	9 (3.4)	148 (3.2)	32 (3.6)	3,386 (3.4)
O	4.3	9 (3.4)	192 (4.2)	58 (6.6)	4,142 (4.2)
P*	2.9	16 (6.0)	345 (7.6)	67 (7.6)	6,679 (6.8)
Q*	5.0	20 (7.5)	295 (6.5)	37 (4.2)	6,908 (7.0)
R	4.3	1 (0.4)	9 (0.2)	0	201 (0.2)
S	1.1	5 (1.9)	93 (2.0)	13 (1.5)	2,129 (2.2)
T*	3.1	12 (4.5)	188 (4.1)	33 (3.7)	3,795 (3.9)
U*	2.1	5 (1.9)	94 (2.1)	19 (2.2)	1,899 (1.9)
V	2.9	1 (0.4)	18 (0.4)	4 (0.5)	275 (0.3)
W	2.9	4 (1.5)	80 (1.8)	10 (1.1)	1,279 (1.3)
X*	5.7	11 (4.1)	169 (3.7)	24 (2.7)	3,304 (3.4)
Y	2.4	2 (0.8)	20 (0.4)	1 (0.1)	449 (0.4)
Z*	8.3	23 (8.6)	398 (8.7)	67 (7.6)	7,980 (8.1)
Centromeric*		9 (3.4)	98 (2.1)	6 (0.7)	2,563 (2.6)
Total	100.0	266 (100)	4,563 (100)	882 (100)	98,231 (100)

*Chromosome segments known to contain at least one contig with a clone that hybridized to a different segment.

Using cytogenetic data to validate a physical map has been performed previously in Drosophila species [25,26]. However, to our knowledge, this is the first time it has been used to anchor the physical map of an insect of agricultural importance. Our method was analogous to performing linkage analysis with the two most terminal BACs in the contigs. Using that approach, 18 of the 19 longest contigs (95%) in the catfish HICF map were validated [17]. Therefore, although the two approaches are not entirely comparable, our results suggest that the Hessian fly map may have a few more errors. Nevertheless, our results also clearly indicate that the Hessian fly assembly was largely valid and that most errors are associated with the contigs containing the greatest number of Qs. Moreover, they demonstrated that the Hessian fly map can be easily improved using FISH as a manual contig-editing tool.

### Genome coverage

To further evaluate the coverage of the assembled contigs, FISH was used to position 70 additional contigs. In these experiments, only one BAC was used as probe, but we avoided invalid contigs by selecting those that had an average of only 0.1 ± 0.1 Qs per contig. These experiments brought the total number of BACs used as probes to 489 and the total number of contigs that had been FISH-mapped to 266 (Table 4). Of these contigs, 257 were assigned to single chromosome segments. Nine contigs hybridized to the pericentromeric heterochromatin of all four chromosomes, and could not be assigned to a single chromosome segment. The relative length of each chromosome segment was used as a measure of genome content so that the amount of coverage provided to each segment could be compared (Table 4). Except for the nucleolar organizing region (NOR), segment E, every chromosome segment contained at least one contig. The subtelomeric segments A, P, and Q, as well as segments B, F, and H, had a slightly greater proportion of contigs than their relative lengths. In the remaining segments, the proportion of contigs and BACs approximated relative length. Thus, the contigs appeared to be evenly distributed. Disregarding the FPC assembly errors previously discovered, and ignoring the centromeric contigs, 95,668 kb of genome length was covered by the FISH mapped contigs. We observed no evidence suggesting that any contig was exclusively associated with the E chromosomes. This coverage therefore represents approximately 60% of the total content of the Hessian fly autosomes and X chromosomes. Moreover, the heterochromatin of these chromosomes is restricted to the centromeres [27]. These contigs therefore clearly appeared to be distributed in the gene rich regions of these chromosomes.

The FPC assembly was developed into a publically available WebFPC archive that shows the BAC clones that were used as probes to physically anchor each contig [21]. Ordering the contigs that are currently grouped into chromosome segments will be one of the most immediate improvements we make to the map. The segments on the autosomes and the long arm of chromosome X1 are expected to provide sufficient resolution to order the contigs using FISH. However, morphologies of the short arm of chromosome X1 and all of chromosome X2 are problematic. We therefore expect that genetic mapping will be necessary to order the contigs in the corresponding segments.

### BAC-end sequence

In order to fully utilize the physical map as a resource in genetic investigations, comparative analyses, and whole genome sequence assembly, we end sequenced the 13,614 fingerprinted BAC clones that were used to generate the physical map. This resulted in 21,814 sequenced BAC ends, 4,708 paired reads, and 13,351,753 bp of high quality bases of Hessian fly genomic DNA [GenBank Trace Archive TI numbers 2136865139–2136875614 and 2136877165–2136888504]. If there were no overlap among the BAC ends, this sequence would represent 8.4% of the Hessian fly genome. The average number of bases sequenced per successfully sequenced BAC end was 764 ± 237. G/C content of all sequenced BACs was relatively low, averaging 33.4 ± 0.03%.

A combination of a custom repeat library and RepBase database [28] was used to identify repetitive DNA sequence. Because the BACs were derived from heterogeneous strains, this analysis required parameters that limit the possibility of confusing alleles with gene families. Therefore, only repetitive sequences that were >40 bp and present in = 5 copies were selected. These criteria made identification of gene families more likely, given the low coverage of BES. Using these settings, we found that 17.91% of the BES contained repetitive elements (Table 5). There was, however, a 1.48% overestimate of repetitive content due to the difficulty of assigning boundaries to different repeat classes. Transposable Elements (TEs) composed only 4.53% of the repetitive DNA and 0.81% of total BES (Table 5). Combined, Helicases, ribosomal DNA (including tRNA, rRNA and 60S and 40S ribosomal proteins), simple sequence repeats (SSRs), and sequence of low complexity composed 15.79% of the repetitive fraction. The largest fraction of repetitive DNA (79.68%) consisted of multigene families and unclassified TEs. This observation was consistent with previous investigations of the Hessian fly salivary gland transcriptome [10,11,29] that discovered over 200 gene families of small putatively secreted proteins with no sequence similarities to other genes in GenBank. In the BES, the most prominent multigene families identified appeared to be tandem copies of the calponin homology domain (an actin binding domain), thrombospondin type1 repeats (which bind and activate TGF-beta), U1 small nuclear ribonucleoprotein C proteins (involved in mRNA splicing), and SMC-hinge family genes (involved in the structural maintenance of chromosomes).

**Table 5:** Repetitive sequence composition of Hessian fly BAC ends.

Repeat class	Repeat type	Total bp	% BES	% Total repetitive
Class I transposable elements		**62,774**	**0.47**	**2.64**
	LTRs	36,337		1.52
	Non-LTRs	17,197		0.72
	Unknown	9,240		0.39
Class II transposable elements		**45,599**	**0.34**	**1.91**
	*hAT*	67		0.00
	*Mos 1*	39,806		1.66
	*mariner*			
	Other	3,664		0.15
	*mariner*			
	Other	2,062		0.08
Simple sequence repeats		107,962	0.81	4.51
Low-complexity DNA		239,182	1.79	10.00
Ribosomal	rRNA	17,249	0.13	0.72
	tRNA	536	0.00	0.02
	protein	3918	0.03	0.16
Helicases		8,862	0.07	0.37
Gene families & unclassified TEs		1,905,881	14.27	79.68
Total Repetitive		2,391,963	17.91	
Total BES		13,351,753		

Within the Class I TEs, LTR retrotransposons were more prominent than the non-LTR retrotransposons (Table 5). The most prominent Class II TE was the *Mos1 mariner*-like DNA transposable element, which accounted for 1.7% of the repetitive fraction. The remaining Class II TEs composed 0.24% of the repetitive sequence, which had sequence similarities to the *Tigger2*, *Blackjack*, *Looper*, *MER45R*, and *Zaphod *TEs. Previous investigation found an abundance of *mariner*-like elements in Hessian fly pericentromeric heterochromatin [30]. Therefore, it was interesting to note that the combined proportions of TEs and low-complexity sequence in the BES (2.6%) was virtually the same as the proportion of the BACs that hybridized to pericentromeric heterochromatin in the FISH experiments that anchored the physical map to the chromosomes (2.4%). These observations were consistent with a low abundance of TEs in the euchromatin of the small Hessian fly genome, and they suggested that the BACs that hybridized to heterochromatin contained one or more TEs. This in turn suggests that seven of the eleven contigs that were initially classified as "invalid-repetitive" (Table 3) would have been classified as "valid" if not for the presence of one or more TEs in the BACs used as probe.

SSRs, which have a wide range of applications [31], formed 0.81% of the total BES and 4.51% of the total repetitive fraction. The di- and tri-nucleotide repeats were 29.1% and 33.4% of the SSRs, respectively, and were the most frequent types of SSRs found in this analysis. The most abundant di- and tri-nucleotide motifs were (TC/GA)n and (TAA/TTA)n, which composed 49.9% of the total di-nucleotides and 43.7% of the total tri-nucleotides, respectively. The value of 'n' ranged from 10 to 56 in the (TC/GA)n motif and from 7 to 76 in the (TAA/TTA)n motif.

To identify putative genic sequences in the BES, both the repeatmasked and unmasked BESs were searched against the NCBI non-redundant database using BLASTX [32]. Of the 20,487 total unmasked BES analyzed, 4,221 had blast hits, of which 2,770 were mapped to gene ontology (GO) terms and 1,847 were annotated. For the repeatmasked BES dataset, 3,639 had BLAST hits, 2,374 were assigned to GO categories and 1,565 were returned with annotations. Among completely sequenced genomes, mosquitoes, which belong to the same suborder (Nematocera) as the Hessian fly, had the highest percentages of the top scoring BLASTX hits (*Aedes aegypti*, 17.3%; *Culex quinquefasciatus*, 15.7%; and *Anopheles gambiae*, 10.7%). Of the total number of reads that had a blast hit, 3,386 hit a conserved protein sequence in an insect genome. In both the repeatmasked and unmasked datasets the largest GO categories comprised genes involved in transcription and cellular communication. For example, genes involved in protein binding, nucleic acid and nucleotide binding, hydrolase activity, transferase and signal transduction activities comprised the largest GO categories (86% of the total) based on the association with gene ontology terms in the molecular function category [see Additional file 1]. Interestingly, fewer annotated sequences were observed in the repeatmasked set for almost all categories. This observation indicated that a proportion of the predicted gene products were derived from the repetitive sequences, which when masked, resulted in lower numbers of annotations. This was also consistent with the earlier observation that multigene families composed a relatively large proportion of the repetitive BES. To examine this further, we unmasked the SSRs so that they were excluded from the repeatmasked set [see Additional file 2]. Again, we observed fewer annotations with that data set than with unmasked BES. Therefore, multigene families and transcripts from transposable elements probably accounted for the observed differences between the two data sets.

## Conclusion

Hessian fly's small genome and the polytene chromosomes provided an excellent opportunity to determine if BAC libraries constructed from heterogeneous strains would unduly limit the mapping abilities of the HICF and FPC technologies. Although less heterogeneity would have been preferred, the results have clearly improved the Hessian fly as an experimental model. A first generation map now exists that includes 266 FPC contigs containing 4,563 BAC clones and their associated BES positioned to 26 segments of the Hessian fly genome. The BES provided new knowledge regarding Hessian fly genome organization, and the mapped BES constitutes an abundant resource for the development of physically mapped DNA markers. We expect that these improvements will be made as genetic investigations focus on the discovery of avirulence genes, the genes that are involved in plant-gall formation, and the mechanisms of chromosome elimination. In the near future, we also expect the map to facilitate the assembly of whole-genome shotgun sequences and the assignment of sequenced scaffolds to chromosome segments.

## Methods

### BAC libraries

Three BAC libraries were utilized in this investigation. Each library was constructed using a separate heterogeneous source of genomic DNA. The Hf library, previously described by Liu *et al*. 2004 [33], was prepared from DNA isolated from a Kansas "Great Plains" (GP) population. To supplement the Hf library, we used clones derived from the MD_Bb library developed by the Clemson University Genomics Institute [34]. Herein referred to as the "CL" library, these BACs were prepared from DNA isolated from an Indiana "L" Hessian fly population. From a third library (Mde), 78 BACs were fingerprinted. Seventy-three of these clones were present in two contigs that were previously developed by chromosome walking [9]. These clones therefore served as an internal control while developing contigs from the fingerprint data using FPC software [19]. The Mde library was constructed with DNA isolated from the Georgia derived "vH13" Hessian fly population as described previously [8]. Plates and filters of all of these clones are available on a cost-recovery basis by request from the Purdue Genomics Center [35].

### DNA fingerprinting

High-information content DNA fingerprints of BACs were obtained using the SNaPshot Primer Extension Kit (Applied Biosystems, CA) as described by Luo *et al*. (2003) [14] with little modification. Each BAC clone was grown in a separate well of a 96-well micro-plate (QIAGEN, CA) containing 150 μl of 2× YT medium (12.5 μg/ml of chloramphenicol). BAC DNA was extracted from each well using the R.E.A.L 96-prep kit following the manufacturer's recommendations (QIAGEN, CA). A QIAvac 96 Manifold, attached to a BioRobot 3000 (QIAGEN, CA), was used to vacuum (-930 mbar) collect the lysate through the QIAfilter into new 96-well blocks. The DNA was then precipitated with iso-propanol, collected by centrifugation, and re-suspended in 40 μl of distilled (dd) H_2_O. The DNA concentration in four wells per plate was then determined by agarose gel electrophoresis. DNA concentration ranged from 50 to 100 ng/μl.

To fingerprint each BAC, 1 μg of each sample was transferred to a separate well of a 96-well plate. This DNA was then restricted at 37°C for 3 h with 4-units of each of five restriction endonucleases (*Eco*RI, *Bam*HI, *Xba*I and *Xho*I and *Hae*III) in 50 μl of a solution containing 5 mM NaCL, 10 mM Tris-Cl (pH 7.9), 1 mM MgCl_2_, 0.1 mM dithiothreitol, 100 μg/μl bovine serum albumin, 0.5 μg/μl DNase free RNase A, and 0.02% β-mercaptoethanol. After the restriction digestions, 10 μl of a solution containing 0.4 μl of the SNaPshot multiplex kit, 7 μl of 33 mM Tris-Cl (pH 9.0), 50 μmoles of NaCl, and 10 μmoles of MgCl_2 _were added directly to the solution in each well. This mixture was incubated at 65°C for 1 h. The labeled DNA was then precipitated and air-dried. To prepare each sample for analysis, each labeled DNA pellet was suspended in 6 μl of Hi-Di Formamide (Applied Biosystems, CA) and 3 μl of ddH_2_O. To this solution, 0.05 μl of an internal size standard (Liz 500; Applied Biosystems, CA) was added. The DNA was then denatured at 95°C and the labeled fragments were separated and sized using an ABI 3730 DNA analyzer.

### Fluorescence in situ hybridization (FISH)

Polytene chromosome preparations and *in situ *hybridizations were performed as previously described [36]. Briefly, BAC DNA (1 μg) was labeled with either biotin- or digoxigenin-conjugated dUTP (Roche) by nick-translation. Hybridizations were performed overnight at 37°C in a solution (10 μl) containing 40–100 ng of denatured probe DNA in 10 μl of hybridization solution (10% dextran sulfate, 2× SSC, 50% deionized formamide, and 10 μg denatured salmon sperm DNA) under a coverslip. Detection was performed using Alexa Fluor 488-conjugated anti-biotin and rhodamine-conjugated anti-digoxigenin (Molecular Probes-Invitrogen). When testing for the co-localization of BACs in the same contig, both labeled BACs (one labeled with biotin and the other labeled with digoxigenin) were hybridized simultaneously to the same polytene chromosome preparations with the expectation that the probes would co-localize if the contigs were genuine. Digital images were taken using UV optics on an ORCA-ER (Hammanmatsu) digital camera mounted on an Olympus BX51 microscope, and MetaMorph (Universal Imaging Corp.) imaging software.

### Chromosome walking

Two BAC contigs were used to test the reliability of the FPC-based Hessian fly contigs. These contigs were constructed by chromosome walking from two markers flanking the Hessian fly *Avirulence *gene, *vH13*. The walk from marker 124 was previously described [9]. The walk from marker 134 is described above (Figure 3). Chromosome walking experiments consisted of BAC library screens using ^32^P-dCTP-labeled probes prepared from markers and BAC-end DNA sequence. Probe labeling and the isolation of BAC-end DNA were performed as described previously [9].

### BAC-end Sequence Analysis

BAC clone DNA was prepared and sequenced in an automated process using a 384-well format. BAC clones were grown in 375 μl of sterile TB supplemented with chloramphenicol (12.5 μg/ml) for 20 hours at 30°C. Alkaline lysis was used to isolate BAC DNA. Sequencing was performed in 7.5 μl volumes containing 0.5 μl of Big Dye Terminator v3.1 (Applied Biosystems), 1.8 μl of Big Dye Terminator v1.1/v3.1 5× buffer (Applied Biosystems), 3 pmoles of primer, and 100 to 250 ng of BAC DNA. Oligonucleotides T7-ZL (TAATACGACTCACTATAGGG) and BES-HR (CACTCATTAGGCACCCCA) were used to prime separate reactions from opposite ends of each BAC template. Those reactions were performed in a GeneAmp PCR System 9700 (Applied Biosystems). Using primer T7-ZL, sequencing reactions underwent 120 cycles of 96°C for 10 s, 45°C for 5 s, and 60°C for 8 min. Using primer BES-HR, sequencing reactions underwent 120 cycles of 96°C for 10 s, 45°C for 5 s, and 52°C for 8 min. The sequencing products were cleaned using ethanol precipitation and then resuspended in 15 μl of ddH2O. Sequences were then determined using a 3730XL Genetic Analyzer (Applied Biosystems).

Analysis of repetitive sequence in BES was performed using a custom repeat database combined with RepBase database version 20061006 [28]. The custom repeat database was constructed using RECON [37]- a *de novo *approach to identify repetitive sequences. PERL scripts were used to select repetitive sequences greater than 40 bp in length and present in 5 or more copies, which were then annotated using BLASTX [32] at e = 10^-5 ^to the NCBI non-redundant database. The annotated repeat database was used as a custom repeat library for RepeatMasker [38]. RepeatMasker version 3.1.9 was used at a default mode with WU-BLAST as the search engine (blastp version 2.0 MP-Washington University) to mask the repetitive sequences in the BESs. Another round of RepeatMasker using the RepBase update 20061006 (RepBase database version 20061006) was also run on the same set of sequences. Results from both runs were combined after manually removing the overlaps. The masked sequences were then categorized into different classes of repetitive sequences.

To identify potential genic sequences and to look for differences between the repeat-masked and unmasked BESs, we used both sets of sequences for GO analysis. The two sets of sequences were used as queries to the NCBI non-redundant database using BLASTX (e = 10^-5^). The BLAST output in the XML format was imported into BLAST2GO (B2G) for GO analysis and functional annotation of gene or protein sequences [39]. The resulting annotations were converted into 'GO-Slim' format and retrieved for the three GO categories (biological process, molecular function and cell component) with an alpha score of at least 0.6 and an ontology depth level of 3.

## Authors' contributions

RA and TB performed BAC fingerprinting, and FISH, and participated in drafting the manuscript. RA performed contig assembly. NG analyzed BES and participated in drafting the manuscript. CZ performed FISH and critically reviewed the manuscript. JPF optimized BAC fingerprinting and supervised its implementation. BS performed BAC-end sequencing and critically reviewed the manuscript. JPF, MSC, and JJS conceived, initiated, and supervised the project. JPF and MSC critically reviewed the manuscript. JJS performed chromosome walking, data analysis, and drafted the manuscript. All authors read and agreed on its final version.

## Supplementary Material

Additional file 1 **Gene ontology (GO) classification of Hessian fly BAC-end sequences**. Unmasked and repeatmasked BAC-end sequence data sets were annotated and assigned to 16 molecular function gene ontology categories. Sequences with simple sequence repeats (SSRs) were included in the repeatmasked data set. The numbers of annotated sequences were greater in the unmasked set than in the repeatmasked set. Click here for file

Additional file 2 **Gene ontology (GO) classification of Hessian fly BAC-end sequences (SSRs unmasked)**. Unmasked and repeatmasked BAC-end sequence data sets were annotated and assigned to 16 molecular function gene ontology categories. Sequences with simple sequence repeats (SSRs) were excluded from the repeatmasked data set. The numbers of annotated sequences were greater in the unmasked set than in the repeatmasked set. Click here for file
