# Supplementary figures and images for: A BAC-based physical map of the Hessian fly genome anchored to polytene chromosomes
Source: BMC Genomics. 2009 Jul 2;10:293. doi: 10.1186/1471-2164-10-293 (PMC2709663; doi:10.1186/1471-2164-10-293)

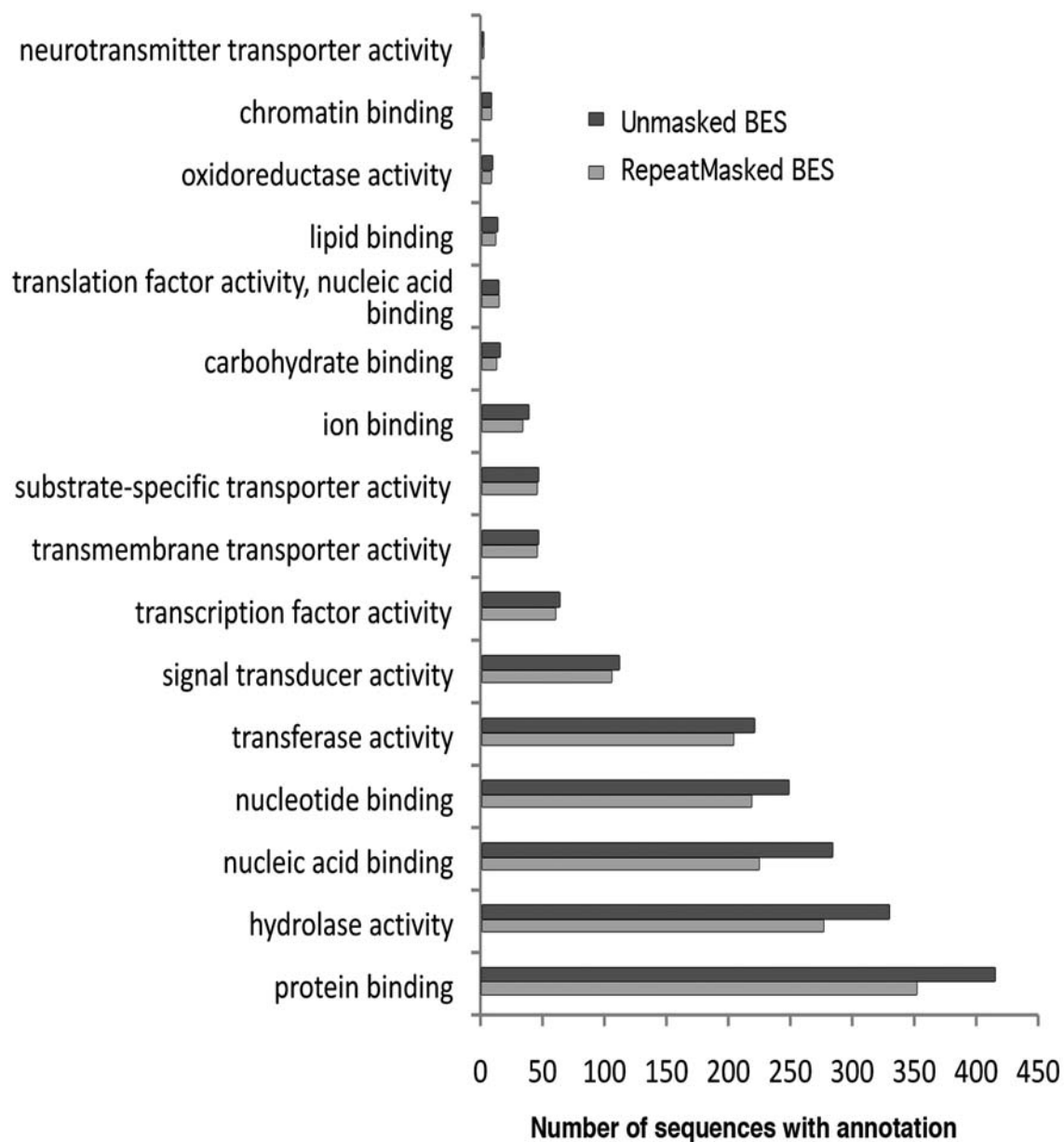

Supplement: Additional file 1 — Gene ontology (GO) classification of Hessian fly BAC-end sequences. Unmasked and repeatmasked BAC-end sequence data sets were annotated and assigned to 16 molecular function gene ontology categories. Sequences with simple sequence repeats (SSRs) were included in the repeatmasked data set. The numbers of annotated sequences were greater in the unmasked set than in the repeatmasked set. [file 1471-2164-10-293-S1.pdf]

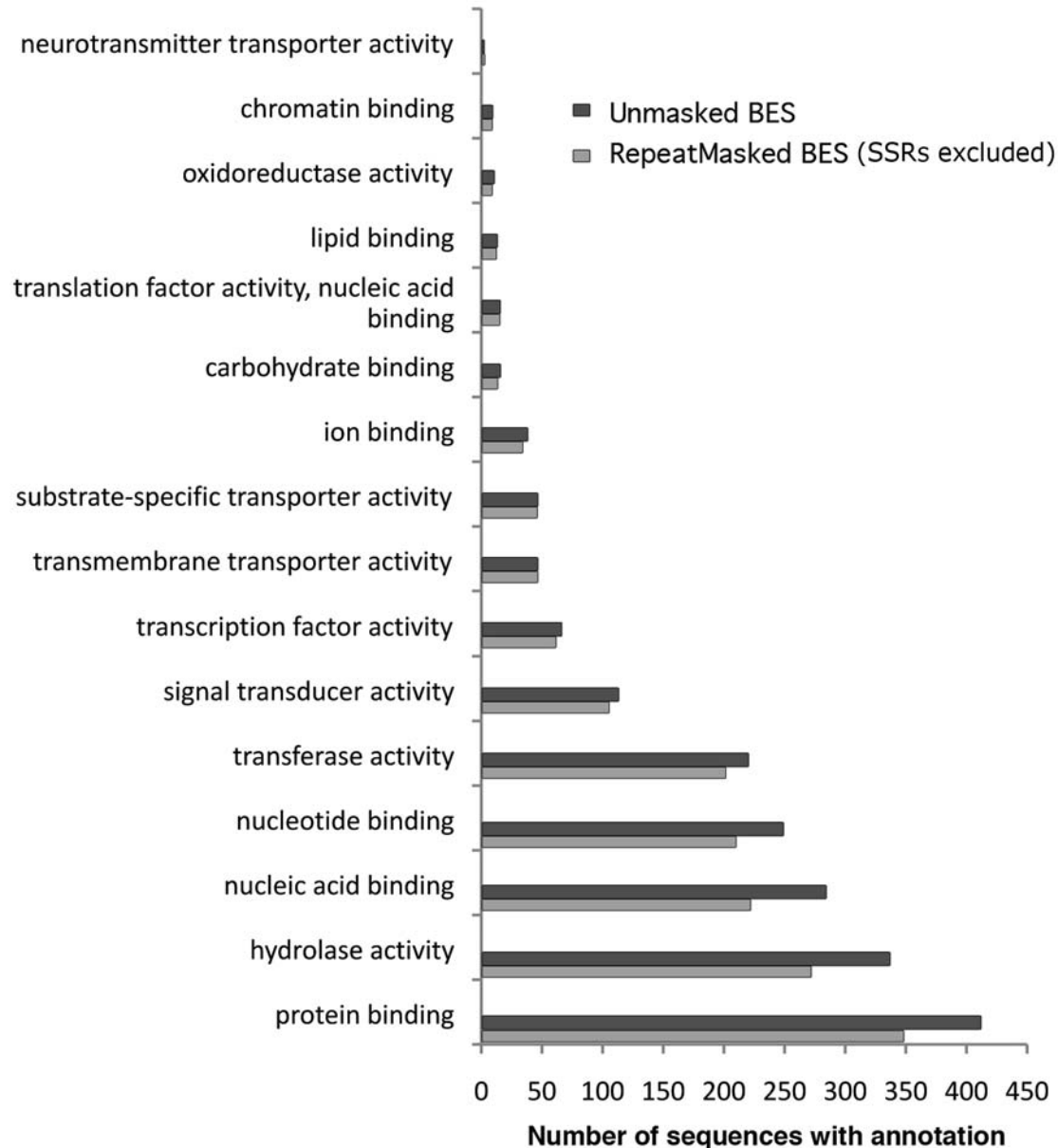

Supplement: Additional file 2 — Gene ontology (GO) classification of Hessian fly BAC-end sequences (SSRs unmasked). Unmasked and repeatmasked BAC-end sequence data sets were annotated and assigned to 16 molecular function gene ontology categories. Sequences with simple sequence repeats (SSRs) were excluded from the repeatmasked data set. The numbers of annotated sequences were greater in the unmasked set than in the repeatmasked set. [file 1471-2164-10-293-S2.pdf]
